# Cross-cultural validation of health literacy measurement tools in Italian oncology patients

**DOI:** 10.1186/s12913-017-2359-0

**Published:** 2017-06-19

**Authors:** Paola Zotti, Simone Cocchi, Jerry Polesel, Chiara Cipolat Mis, Donato Bragatto, Silvio Cavuto, Alice Conficconi, Carla Costanzo, Melissa De Giorgi, Christina A. Drace, Federica Fiorini, Laura Gangeri, Andrea Lisi, Rosalba Martino, Paola Mosconi, Angelo Paradiso, Valentina Ravaioli, Ivana Truccolo, Paolo De Paoli, ICPEG, Marta De Conti, Francesca Agostina Masutti, Silvia Flora, Sara Francescon, Valentina Tomasi, Carla Bagnacani, Angela Nardi, Cristina Nanni, Petrina Mariana Braghesiu

**Affiliations:** 1 Scientific Directorate, CRO Aviano National Cancer Institute – IRCCS, Aviano, PN Italy; 2 Medical Library, Arcispedale Santa Maria Nuova – IRCCS, Reggio Emilia, Italy; 3 Unit of Cancer Epidemiology, CRO Aviano National Cancer Institute, IRCCS, Aviano, PN Italy; 4 Scientific and Patients Library, CRO Aviano National Cancer Institute – IRCCS, Aviano, PN Italy; 5 Health Sciences Library, Azienda Ospedaliero-Universitaria-Ferrara, Ferrara, Italy; 6 Infrastructure Research and Statistics-Scientific Directorate, Arcispedale Santa Maria Nuova – IRCCS, Reggio Emilia, Italy; 7 Public Relation, Media and Communication Office, Istituto Scientifico Romagnolo per lo Studio e la Cura dei Tumori (IRST)-IRCCS, Meldola, FC Italy; 8 Psycho-Oncology Unit, National Cancer Research Center-Istituto Tumori “Giovanni Paolo II” IRCCS, Bari, Italy; 9 Psycho-Oncology Unit, Veneto Institute of Oncology IOV- IRCCS, Padua, Italy; 10 Scientific Directorate, Veneto Institute of Oncology IOV – IRCCS, Padua, Italy; 11 Communication & Welcome Area, Azienda Ospedaliero-Universitaria, Ferrara, Italy; 12 Clinical Psychology Unit, IRCCS National Cancer Institute of Milan, Milan, Italy; 13 Laboratory for Medical Research and Consumer Involvement, IRCCS Istituto di Ricerche Farmacologiche Mario Negri, Milano, Italy; 14 Experimental Medical Oncology-Oncology Dept, National Cancer Research Center-Istituto Tumori “Giovanni Paolo II” IRCCS, Bari, Italy; 15 Scientific and Patients Library-Scientific Directorate, CRO Aviano National Cancer Institute – IRCCS, via Gallini 2, 33081 Aviano, PN Italy

**Keywords:** Health literacy, Measurement, Validation, Cross-sectional studies, Cross-cultural adaptation, Neoplasms, Patient education & empowerment, Questionnaires, Italy

## Abstract

**Background:**

The aim of this study was to assess the psychometric characteristics of four Health Literacy (HL) measurement tools, viz. Newest Vital Sign (NVS), Short Test of Functional Health Literacy in Adults (STOFHLA), Single Item Literacy Screener (SILS) and Single question on Self-rated Reading Ability (SrRA) among Italian oncology patients.

**Methods:**

The original version of the tools were translated from the English language into Italian using a standard forward-backward procedure and according to internationally recognized good practices. Their internal consistency (reliability) and validity (construct, convergent and discriminative) were tested in a sample of 245 consecutive cancer patients recruited from seven Italian health care centers.

**Results:**

The internal consistency of the STOFHLA-I was Chronbach’s α=0.96 and that of NVS-I was α=0.74. The STOFHLA-I, NVS-I, SILS-I and SrRA-I scores were in a good relative correlation and in all tools the discriminative known-group validity was confirmed. The reliability and validity values were similar to those obtained from other cultural context studies.

**Conclusion:**

The psychometric characteristics of the Italian version of NVS, STHOFLA, SILS and SrRA were found to be good, with satisfactory reliability and validity. This indicates that they could be used as a screening tool in Italian patients. Moreover, the use of the same cross-cultural tools, validated in different languages, is essential for implementing multicenter studies to measure and compare the functional HL levels across countries.

**Electronic supplementary material:**

The online version of this article (doi:10.1186/s12913-017-2359-0) contains supplementary material, which is available to authorized users.

## Background

In 2003, the National Assessment of Adult Literacy defined Literacy as “the ability to use printed and written information to function in society, to achieve one's goals, and to develop one's knowledge and potential” [1]. “Literacy is content and setting specific. An individual may have adequate understanding of material with familiar content, but struggle to comprehend information with unfamiliar vocabulary and concepts” [2]. The operational definition of Health Literacy (HL), developed for the National Library of Medicine, describes HL as “the degree to which individuals have the capacity to obtain, process and understand basic health information and services needed to make appropriate health decisions” [3]. Numerous studies have shown that an inadequate HL level is associated with inadequate understanding of written information, lack of medical information, less use of preventive measures, lower medication compliance, impaired health care knowledge, poor health-related outcomes and higher health care costs [4]. Appropriate and valid HL measurement tools are essential in research to evaluate the effect of HL on several health outcomes, such as healthcare use, and the effectiveness of preventive healthcare and targeted interventions [5]. Furthermore, they are useful in clinical practice to improve communication with patients. In the literature, several tools have been proposed in research and clinical settings [6] with different characteristics, validity and reliability history. Most of these tools have been developed in the Anglo-Saxon context and a cross-cultural process of implementation in other languages and cultures is important. This issue is a crucial aspect for the multicenter collaborative Italian Cancer Patient Education Group project whose aims are: to map patient education (PE) activities in the different Italian cancer research and care institutes [7], to survey the unmet information and communication needs, and to implement PE programs and activities with the involvement of patient representatives in each phase [8]. Thus, validating the tools for measuring functional HL among Italian cancer patients is a key step of our research, because knowing the level of functional HL is essential for all PE activities.

To date, several instruments have been proposed to measure HL, but none of them have been sufficiently validated for the Italian population. The Newest Vital Sign (NVS), the Short Test of Functional Health Literacy in Adults (STOFHLA) and the Single Item Literacy Screener (SILS) are commonly used in HL research. The Italian Cancer Patient Education Group carried out this study, aiming to translate and to cross-culturally adapt the original English version of these HL measurement tools for Italian oncology patients.

## Methods

### Participants

An observational cross-sectional study was conducted within the Italian Cancer Patient Education Group between November 2015 and February 2016. The data was collected from patients attending seven different hospitals in Italy, three in Northern Italy, three in the Centre and one in the South. Consecutive adult cancer patients, aged 18–65 years, were enrolled after being approached by trained research assistants in the waiting rooms of the oncology, surgery or day-hospital wards. Patients were included if they were cognitively able to understand and complete the survey. Language proficiency and cognitive functioning was judged by the research assistant upon inclusion. According to study protocol, the minimum sample size was calculated as at least 5 patients for each questionnaire item of the longest questionnaire [9]. Therefore, for our study aims, at least 200 patients were required, based on STOFHLA (40 items). Since the catchment area was different for each centre, there was no competitive recruitment.

### Ethics

The study was approved by the Ethics Committee of each participating center, eg: CRO Aviano National Cancer Institute -IRCCS, Aviano; Arcispedale Santa Maria Nuova - IRCCS, Reggio Emilia; Azienda Ospedaliero-Universitaria Ferrara, Ferrara; Istituto Scientifico Romagnolo per lo Studio e la Cura dei Tumori (IRST)- IRCCS, Meldola(FC); Istituto Tumori “Giovanni Paolo II” IRCCS, Bari; Veneto Institute of Oncology IOV- IRCCS, Padua; IRCCS National Cancer Institute of Milan. The patients received detailed information about the aims and procedures of the study, and were enrolled once their written informed consent was obtained according to international standards.

### Measurement tools

The study included four Health Literacy measurement tools:

- The NVS, developed by Weiss et al. [10], is a verbally administered 6-item tool that asks about information contained in a standard ice cream nutrition label. It requires the ability to interpret the food table, and to answer six questions related to health. The NVS was developed as a rapid and accurate HL screening test. The average time taken to conduct this test is about 6 min.
- The STOFHLA, developed by Baker et al. [11], is a 40-item questionnaire divided into two sections. The first section includes four verbal numeracy questions, requiring interpretation of medication instructions, test results and an appointment date. The second section includes 36 self-administered items regarding the preparation for a radiological examination (passage A) and a health administrative rights management section (passage B). The compilation of this test requires an average of about 12 min.
- The SILS, developed by Morris et al. [12], is a single-item test designed to measure the need for help with reading health-related materials. The item is administered verbally: “How often do you need to have someone help you when you read instructions, pamphlets, or other written material from your doctor or pharmacy?” The patient is asked to choose one of the following answers on a 5-point Likert scale: Never, Rarely, Sometimes, Often, Always.
- The SrRA, developed by Jeppesen et al. [13], is a single- item test related to self-rated reading ability. The item is administered verbally: “How would you rate your ability to read?” A participant is asked to choose one of the following answers on a 5- point Likert scale: Excellent/ Very good, Good, Okay, Poor, Terrible/Very poor.

### Translation

The translation and cross-cultural adaptation of the original English SILS, NVS, STOFHLA and SrRA, were performed using a standard forward-backward procedure and according to recognized good practices [14, 15]. A pilot study was conducted with 15 patients to assess any ambiguous statements or questions and to make sure the Italian forms were understandable. The final translation form was refined after the feedback from the pilot study and after an agreement among the research group leaders.

### Procedures

During the survey, the translated tools were administered consecutively in the following order: SILS-I, SrRA-I, STOFHLA-I and NVS-I. Since SILS-I and SrRA-I investigate the self-perception of reading ability, they were administered before the STOFHLA-I and NVS-I to avoid any influence on patients’ awareness of reading and numeracy skills. The administration process was standardized among the centers and the interviewers were trained centrally.

The research assistants administered the HL measurement tools to all eligible and consenting patients and recorded their responses. Demographic data such as age, gender, educational level and marital status were collected during the interview.

### Statistical analysis

Reliability analyses were conducted for STOFHLA-I and NVS-I using Cronbach’s alpha, which represent the internal consistency of the items. To estimate the test-retest reliability, the split-half procedure was applied using the equal-length Spearman-Brown formula.

Construct validity was also conducted by computing correlations (Spearman coefficient) between NVS-I and STOFHLA-I, and among STOFHLA-I sections. Known-group validity was tested comparing NVS-I and STOFHLA-I mean scores across age groups, educational level, sex, marital status and geographic area using the analysis of variance (ANOVA). Statistical significance was claimed for *p*<0.05. All analyses were performed using SAS 9.2 software.

## Results

### Participants

A total of 248 patients were enrolled. Three patients were not able to complete the survey and were excluded from the analysis, thus leaving 245 eligible patients. Patients were aged 18–65 years (median: 54 years) and they were predominantly females (*n*=153; 62.4%) (Table 1). The majority had a high school education level (i.e. 11–13 years of schooling; n=124, 50.6%), whereas only 16 patients (6.5%) reported <6 years of schooling. Patient answers to SILS-I and SrRA-I questionnaires and the score of the NVS-I and STOFHLA-I items that were completed correctly are reported in Table 2.

**Table 1:** Socio-demographic characteristics of 245 enrolled cancer patients

Characteristics	N	(%)
Sex		
Male	92	(37.6)
Female	153	(62.4)
Age (years)		
18–45	54	(22.0)
46–49	35	(14.3)
50–54	49	(20.0)
55–59	49	(20.0)
60–65	58	(23.7)
Median (min-max)		54 (18–65)
Education		
None/Primary school	16	(6.5)
Secondary school	63	(25.7)
High school	124	(50.6)
University or higher	42	(17.1)
Marital status		
Single/Widowed/Divorced	64	(26.1)
Married/Cohabitant	181	(73.9)
Geographic area		
Northern Italy	101	(41.2)
Central Italy	114	(46.5)
Southern Italy	30	(12.2)

**Table 2:** Patient answers to SILS-I and SrRA-I questionnaires and score of NVS-I and STOFHLA-I items completed correctly

Characteristics	N	(%)
Single Item Literacy Screener (SILS-I)		
Never	61	(24.9)
Rarely	81	(33.1)
Sometimes	74	(30.2)
Often	20	(8.2)
Always	9	(3.7)
Self-rated Reading Ability (SrRA-I)		
Excellent	60	(24.5)
Good	112	(45.7)
Adequate	69	(28.2)
Low	4	(1.6)
Very low	0	(0.0)
NVS-I		
0–1	30	(12.2)
2–3	70	(28.6)
4–5	80	(32.7)
6	65	(26.5)
STOFHLA-I		
Numeracy items completed correctly		
0–1	8	(3.3)
2	22	(9.0)
3	54	(22.0)
4	161	(65.7)
Reading items completed correctly		
Passage A^a^		
0–8	16	(6.5)
9–12	16	(6.5)
13–14	36	(14.7)
15–16	177	(72.2)
Passage B^b^		
0–10	29	(11.8)
11–18	75	(30.6)
19–20	141	(57.6)

^a^Passage A includes 16 items regarding the preparation for a radiological examination

^b^Passage B includes 20 items regarding health administrative rights

### Reliability

Chronbach’s alpha coefficient was 0.96 for the overall STOFHLA-I, 0.45 for STOFHLA-I/Numeracy, and 0.96 for STOFHLA-I/Reading sections. The Spearman correlation between Numeracy and Reading was 0.38. The internal consistency of the NVS-I was 0.74. Test-retest reliability was evaluated through the split- half procedure, reporting satisfactory results for both the STOFHLA-I (*r*-Spearman=0.59) and NVS-I (*r*- Spearman=0.65).

### Validity

Construct validity was evaluated by computing discriminative known-group validity analysis as reported in Table 3, and criterion validity by correlation between the tool analyses (Table 4). Significant differences in STOFHLA-I, NVS-I, SILS-I and SrRA-I scores were found between patients of different age groups (*p*<0.05), educational level (*p*<0.01), and geographic area (*p*<0.01). For all tools, no relationship was found with sex or marital status. Given the lack of a validated tool for the assessment of HL in Italy, external validity was evaluated by correlating each HL instrument with the other three.

**Table 3:** Mean score and standard deviation (STD) of NVS-I and STOFHLA-I according to selected patient characteristics

Patient characteristics	NVS-I	STOFHLA-I
	Mean (std)	Mean (std)
Sex		
Male	3.9 (1.8)	33.8 (9.0)
Female	3.9 (1.9)	35.3 (7.3)
ANOVA^a^	*p*=0.95	*p*=0.10
Age (years)		
<45	4.2 (1.7)	37.2 (4.2)
45-49	4.0 (1.8)	36.1 (6.8)
50-54	4.0 (1.8)	35.1 (7.4)
55-59	4.2 (1.7)	35.8 (6.4)
≥60	3.3 (2.1)	30.5 (11.1)
ANOVA^a^	*p*=0.02	*p*<0.01
Education		
None/Primary school	2.6 (1.9)	26.1 (13.6)
Secondary school	3.0 (1.9)	30.9 (10.0)
High school	4.1 (1.7)	36.5 (5.2)
University or higher	5.1 (1.3)	38.8 (1.7)
ANOVA^a^	*p*<0.01	*p*<0.01
Marital status		
Single/Widowed/Divorced	3.9 (1.8)	34.4 (8.2)
Married/Cohabitant	3.8 (2.0)	35.8 (7.3)
ANOVA^a^	*p*=0.79	*p*=0.16
Geographic area		
Northern Italy	3.7 (1.7)	35.4 (7.3)
Central Italy	4.2 (1.9)	35.4 (7.7)
Southern Italy	3.2 (1.9)	30.3 (10.1)
ANOVA^a^	*p*<0.01	*p*<0.01

^a^Including items for sex, age, education, marital status and geographic area

**Table 4:** Mean score and standard deviation (STD) of NVS-I and STOFHLA-I according to SILS-I and SrRA-I

Patient characteristics	NVS-I	STOFHLA-I
	Mean (std)	Mean (std)
Single Item Literacy Screener (SILS-I)		
Never	4.2 (1.7)	35.2 (7.0)
Rarely	4.0 (1.8)	36.0 (6.7)
Sometimes	3.6 (1.9)	35.0 (7.5)
Often/Always	3.6 (2.1)	29.8 (12.0)
ANOVA	*p*=0.06	*p*<0.01
Self-rated Reading Ability (SrRA-I)		
Excellent	4.9 (1.5)	38.2 (2.1)
Good	3.9 (1.8)	35.0 (7.1)
Adequate	3.1 (1.9)	31.2 (10.9)
Low	3.8 (1.5)	38.0 (0.8)
ANOVA	*p*<0.01	*p*<0.01

The STOFHLA-I reported a good correlation with NVS-I (*r*=0.58), and the score demonstrated a significant positive correlation with the two questionnaires related to reading ability SILS-I (*p*<0.01) and SrRA-I (*p*<0.01). The NVS-I score showed a significant correlation with SrRA-I (*p*<0.01), and borderline with SILS-I score (*p*=0.06). The correlation between SILS-I and SrRA-I was significant (< *p*=0.01).

## Discussion

The purpose of this study was to translate HL measurement tools into Italian, assess their cross content validity, and examine their validity and reliability in a sample of oncology patients. Our results showed that the instruments were sufficiently reliable in the Italian culture, showing psychometric properties similar to those reported in other trans-cultural studies.

The internal consistency value of 0.74 of the NVS-I was good and comparable to data obtained in other cultural settings. Indeed, the values reported in other cultures were: 0.74 in United Kingdom [16]; 0.72 in Japan [17]; 0.76 in The Netherlands [5]; and 0.76 in English-speaking Americans [10]. Slightly lower values was reported in a USA Spanish-speaking group (*α*=0.69) [10]. The internal consistency of the STOFHLA-I was very good for all items included. The instrument measures both text comprehension (reading) and numeracy skills. Once the Reading and the Numeracy sections were evaluated separately, only Reading showed good consistency, while Numeracy gave a poorer result. This was also reported in the original validation paper by Baker and colleagues [11], and was even lower in the German language version in Switzerland (α=0.33) [18]. The reason for the weakness of the STOFHLA-I/Numeracy internal consistency could be due to the fact that the few items of these sections, only four, may assess non-homogeneous aspects of HL.

The validity was assessed by the correlations within and between the HL tools. In the analysis of criterion-related (convergent) validity, the score of each instrument was compared with all the others. The STOFHLA-I total score reported a good correlation with the total NVS-I, with the SILS-I and the SrRA-I. This suggests that one dimension of the instrument correlates appreciably with other instruments investigating the same postulated area.

The correlation that we found between the NVS-I and the STOFHLA-I (*r*=0.58) was analogous to the Iraqi version (*r*=0.51) [19]. Also, it was comparable to the correlations between the NVS and the similar Test of Functional Health Literacy in Adults (TOFHLA) used in Weiss’ USA version (*r*=0.59) [10] and Rowlands’ UK version (*r*=0.49) [16]. The total NVS-I score did not demonstrate a significant correlation with the SILS-I score. It was once again analogous to the result of the Iraqi NVS version. The NVS-I and STOFHLA-I demonstrated a significant positive correlation with the SrRA-I single item “How would you rate your ability to read?” In our sample, this would suggest that there is a match between what patients think they know and what they really are able to do. Also, Jeppesen et al. [13] reported a similar observation, concluding that “self-rated reading ability was the single most reliable predictor of limited HL of the predictors tested”.

The four tools under investigation showed a satisfactory discriminatory capacity. For all the instruments, the discriminative known-group validity was confirmed, showing significant correlations with age and educational level. Conversely, no correlation was found with gender or marital status.

Some limitations have to be acknowledged. First, the lack of a validated questionnaire for health literacy in Italian did not allow testing external validity through the comparison with a gold standard. Nonetheless, we cross-tested the four instruments, finding good agreement. Further, the oncologic setting may have impacted on patients’ participation and on survey completion. Indeed, the approached patients may have had cancer-related fatigue, which impacted on refusal rate, and survey could have been interrupted by patient call to visit or treatment. However, only three patients refused study participation (refusal rate: 1.2%) and three additional patients (1.2%) interrupted the survey (1 for fatigue and 2 for calling to visit/treatment). The inclusion of study centers from all parts of Italy should be accounted among the study strengths. Indeed, this guaranteed that study patients were a representative sample of the Italian population. This also allowed the evaluation of the geographic variation in health literacy. Sample size should also be acknowledged among study strengths, since it guaranteed an adequate power.

### Implications for practice and further research

The psychometric characteristics of the Italian translations of NVS, S-THOFLA, SILS and SrRA were found to be acceptable with satisfactory reliability and validity, which indicate that they can be used as a screening tool in Italian patients. The findings are useful for planning and re-building health services with a patient-centered approach.

Moreover, the use of the same cross-cultural tools, validated in different languages, is essential for implementing multicenter studies to measure and compare the functional HL levels across countries.

## Conclusions

This study examined cross-culturally adapted tools for assessing objective HL in the Italian oncology context. The psychometric characteristics of the Italian translations of STOFHLA, NVS, two single-item SILS and SrRA were found to be good. The reliability and validity values were similar to those obtained from other cultural context psychometric studies. It means that they can be successfully used as a screening tool in Italian patients. The findings are useful for planning and re-building health services with a patient-centered approach. Moreover, the use of the same cross-cultural tools, validated in different languages, is essential for implementing multicenter studies to measure and compare the functional HL levels across countries.

## Additional files

Additional file 1: Newest Vital Signal(SILS)_Italian version. (PDF 301 kb)

Additional file 2: Single Item Literacy Screener-Italian version. (PDF 73 kb)

Additional file 3: Single question on Self-rated Reading Ability(SrRA)_Italian version. (PDF 72 kb)

Additional file 4: Short-Text of Functional Health Literacy in Adults(S-TOFHLA) Numeracy Section_Italian version. (PDF 729 kb)

Additional file 5: Short-Text of Functional Health Literacy in Adults(S-TOFHLA) Reading Section_Italian version. (PDF 110 kb)

Additional file 6: Italian Cancer Patients Education Group(ICPEG)_investigators research. (DOCX 10 kb)
