# Supplementary material for: Cross-cultural validation of health literacy measurement tools in Italian oncology patients
Source: BMC Health Serv Res. 2017 Jun 19;17:410. doi: 10.1186/s12913-017-2359-0 (PMC5477151; doi:10.1186/s12913-017-2359-0)
Supplement: Supplementary file 1 — Newest Vital Signal(SILS)_Italian version. (PDF 301 kb) [file 12913_2017_2359_MOESM1_ESM.pdf]

TABELLA NUTRIZIONALE

Gelato alla nocciola

Confezione da 4 porzioni

1 porzione = 100 g

Ingredienti: latte scremato, zucchero, pasta di nocciola (7%), destrosio, sciroppo di glucosio, panna, burro, olio di arachidi, stabilizzanti (gomma di guar, carragenina), sale, aromi naturali.

**INFORMAZIONI NUTRIZIONALI**	**Per 100 g**	**% AR* per 100 g**
Valore energetico	1046 kJ<br>250 kcal (calorie)	13%
Grassi	12 g	17%
di cui<br>- acidi grassi saturi	9 g	45%
Carboidrati	30 g	12%
di cui<br>- zuccheri	23 g	26%
Fibre	0 g	
Proteine	5 g	10%
Sale	0,05 g	1%

* Assunzioni di riferimento di un adulto medio (8400 kJ / 2000 kcal [calorie]).

DOMANDE:

1. Se lei mangia l'intera confezione, quante calorie mangerà?
Tenga presente che nella tabella le calorie sono indicate come kcal (leggi "kappacal")
risposta esatta: 1000/ 1000 calorie / 1000 kcal

2. Se le è permesso mangiare 60 grammi di carboidrati come spuntino, quanto gelato può mangiare?
risposta esatta: 2 porzioni / fino a 2 porzioni / (al) massimo 2 porzioni / non più di 2 porzioni / mezza (metà) confezione / fino a mezza (metà) confezione / (al) massimo mezza (metà) confezione / non più di mezza (metà) confezione / 200 grammi / fino a 200 grammi / (al) massimo 200 grammi / non più di 200 grammi

3. Il suo medico le consiglia di ridurre la quantità di acidi grassi saturi nella sua dieta. Lei mangia solitamente 42 g di acidi grassi saturi al giorno, compresa una porzione di gelato. Se smettesse di mangiare gelato, quanti grammi di acidi grassi saturi mangerebbe ogni giorno?
risposta esatta: 33 è l'unica risposta esatta

4. Tenga presente che nella tabella le calorie sono indicate come kcal (leggi "kappacal"). Se lei mangia abitualmente 2500 calorie al giorno, quale percentuale di queste calorie mangia con una porzione di questo gelato?
risposta esatta: 10% o 1/10 (un decimo).

5. Immagini di essere allergico a queste sostanze: penicillina, arachidi, guanti in lattice e punture d'ape. È sicuro per lei mangiare questo gelato?
risposta esatta: no/non sicuro

6. (*Chiedere solo se il paziente risponde "no" alla domanda 5*): perché no?
risposta esatta: perché contiene olio di arachidi.
