# Supplementary material for: Cross-cultural validation of health literacy measurement tools in Italian oncology patients
Source: BMC Health Serv Res. 2017 Jun 19;17:410. doi: 10.1186/s12913-017-2359-0 (PMC5477151; doi:10.1186/s12913-017-2359-0)
Supplement: Supplementary file 2 — Single Item Literacy Screener-Italian version. (PDF 73 kb) [file 12913_2017_2359_MOESM2_ESM.pdf]

SINGLE ITEM LITERACY SCREENER – I

(N. S. Morrys et al. 2006; versione Italiana 2016)

1. **<u>Quanto spesso</u> lei ha bisogno che qualcuno la aiuti quando legge istruzioni, opuscoli o altro materiale scritto di argomento sanitario?**

Possibili risposte:

1. mai
2. raramente
3. ogni tanto
4. spesso
5. sempre
