# Supplementary material for: Cross-cultural validation of health literacy measurement tools in Italian oncology patients
Source: BMC Health Serv Res. 2017 Jun 19;17:410. doi: 10.1186/s12913-017-2359-0 (PMC5477151; doi:10.1186/s12913-017-2359-0)
Supplement: Supplementary file 3 — Single question on Self-rated Reading Ability(SrRA)_Italian version. (PDF 72 kb) [file 12913_2017_2359_MOESM3_ESM.pdf]

Single question on Self-rated Reading Ability – I

Domanda di autovalutazione della capacità di lettura

(K. M. Jeppesen et al. 2009; versione Italiana 2016)

1. **Come valuta la sua capacità di leggere?**

Possibili risposte:

1. molto buona, o ottima
2. buona
3. discreta
4. scarsa
5. molto scarsa, o pessima
