# Supplementary material for: Cross-cultural validation of health literacy measurement tools in Italian oncology patients
Source: BMC Health Serv Res. 2017 Jun 19;17:410. doi: 10.1186/s12913-017-2359-0 (PMC5477151; doi:10.1186/s12913-017-2359-0)
Supplement: Supplementary file 4 — Short-Text of Functional Health Literacy in Adults(S-TOFHLA) Numeracy Section_Italian version. (PDF 729 kb) [file 12913_2017_2359_MOESM4_ESM.pdf]

S-TOFHLA - Numeracy

(R. M. Parker et al. 1995; versione Italiana 2016)

1.

Eritrocina

600 mg

12 compresse

Prendere una compressa per bocca ogni 8 ore.

Domanda: Se prende la prima compressa alle 7:00 di mattina, quando deve prendere la compressa successiva?

risposta esatta: 'alle 15:00' / 'alle 3 del pomeriggio'

2.

Il livello normale di zucchero nel sangue a digiuno è inferiore a 126.

Il Suo livello di zucchero nel sangue oggi a digiuno è pari a 136.

Domanda: Se il <u>suo</u> valore a digiuno fosse questo, il suo livello di zucchero nel sangue sarebbe normale oggi?

risposta esatta: 'no' /'non normale'

3.

APPUNTAMENTO

REPARTO: Diabetologia POSIZIONE: terzo piano

GIORNO: Giovedì DATA: 2 aprile ORE: 10.20

DEVE PORTARE CON SÉ LA SUA TESSERA SANITARIA

Domanda: Quand'è il Suo prossimo appuntamento?

risposta esatta: '2 aprile' / 'Giovedì, 2 aprile'

4.

Levodopa + Benserazide

200 mg + 50 mg

50 compresse

Prendere una compressa a stomaco vuoto, 30 minuti prima dei pasti o un'ora dopo i pasti.

Domanda: Se pranza alle 12.00 e ha intenzione di prendere la compressa dopo pranzo, a che ora deve prenderla?

risposta esatta: 'alle 13:00' / 'dopo le 13:00'
