# Supplementary material for: Cross-cultural validation of health literacy measurement tools in Italian oncology patients
Source: BMC Health Serv Res. 2017 Jun 19;17:410. doi: 10.1186/s12913-017-2359-0 (PMC5477151; doi:10.1186/s12913-017-2359-0)
Supplement: Supplementary file 5 — Short-Text of Functional Health Literacy in Adults(S-TOFHLA) Reading Section_Italian version. (PDF 110 kb) [file 12913_2017_2359_MOESM5_ESM.pdf]

S-TOFHLA - Reading

(R. M. Parker et al. 1995; versione Italiana 2016)

A.

Il Suo medico l'ha mandata a fare una radiografia _____________.

a. allo stomaco
b. al diabete
c. ai punti
d. ai germi

Lei deve avere lo stomaco ____________ quando viene per fare la ____________.

a. asmatico
b. vuoto
c. incesto
d. anemico

a. calligrafia
b. tipologia
c. ortografia
d. radiografia

La radiografia _______________ da 1 a 3 ___________ per essere fatta.

a. richiederà
b. visualizzerà
c. ripeterà
d. guarderà

a. letti
b. teste
c. ore
d. diete

IL GIORNO PRIMA DELLA RADIOGRAFIA.

Per cena, mangi solo ______________ quantità di frutta, _________ e marmellata,

a. una piccola	a. pale
b. un brodo	b. panno
c. un attacco	c. pane
d. una nausea	d. pari

con caffè o tè.

Dopo ______________ , Lei non deve ______________ o bere

a. minuto	a. mandare
b. mezzanotte	b. mangiato
c. durante	c. bevuto
d. prima	d. mangiare

assolutamente _________ fino a dopo aver _________ la radiografia.

a. mente	a. sei
b. niente	b. fai
c. ogni	c. fatto
d. gente	d. era

IL GIORNO DELLA RADIOGRAFIA.

Non mangi a _________________ .

a. appuntamento
b. ambulatorio
c. colazione
d. clinica

Non ____________ , neanche ____________ .

a. beffi	a. cuore
b. beva	b. respiro
c. bendi	c. acqua
d. balli	d. cancro

Se ha qualche ____________ , chiami _____________di Radiologia al numero 02 923111.

a. risposta	a. il reparto
b. esercizio	b. la distorsione
c. apparato	c. la farmacia
d. domanda	d. il mal di denti

B.

Dichiaro di fornire informazioni corrette per _____________ se ho diritto all'Esenzione

a. capelli
b. vestire
c. valutare
d. dolore

dal ticket.

Io _________ di fornire all'Azienda Sanitaria le informazioni per _______________

a. dichiaro
b. esploro
c. invio
d. ottengo

a. nascondere
b. rischiare
c. rilasciare
d. verificare

qualsiasi dichiarazione fornita in questo __________________ e autorizzo

a. enfisema
b. modulo
c. fegato
d. globulo

________________ ad effettuare eventuali controlli. Sono ________________ che

a. l'infiammazione
b. la religione
c. il ferro
d. l'Azienda Sanitaria

a. studioso
b. divertito
c. consapevole
d. stabilito

dovrò segnalare all'Azienda Sanitaria qualsiasi __________________ nella mia situazione

a. cambiamento
b. ormone
c. antiacido
d. caricamento

entro ________ (10) giorni da quando __________ il cambiamento stesso.

a. tre	a. avvelena
b. uno	b. avviene
c. cinque	c. avvilisce
d. dieci	d. avviti

Sono consapevole _____________ se **NON** condivido la ________________ presa nel mio

a. qui	a. coniugale
b. cui	b. occupazione
c. che	c. adulta
d. chi	d. decisione

caso dall'Azienda Sanitaria, in seguito ad un controllo, ho _____________ ad un colloquio.

a. diretto
b. rovescio
c. curvo
d. diritto

Posso _______________ il colloquio scrivendo o _______________ all'Azienda Sanitaria.

a. richiedere	a. tergiversando
b. rifiutare	b. leggendo
c. omettere	c. telefonando
d. aggiustare	d. odorando

Se Lei ____________ l'esenzione dal ticket per un ______________ della famiglia,

a. vuota	a. membro
b. vuole	b. storia
c. copre	c. peso
d. disegna	d. cintura

dovrà compilare e _______________ un altro modulo.

a. rilassare
b. rompere
c. inalare
d. firmare

_____________ , utilizzeremo

a. Da
b. Se
c. Tuttavia
d. Poiché

______________ presente su questo modulo per stabilire la Sua ________________ .

a. il polmone
b. la data
c. il pasto
d. il pelvico

a. ipoglicemia
b. idoneità
c. osteoporosi
d. schizofrenia
