# Supplementary material for: Cross-cultural validation of health literacy measurement tools in Italian oncology patients
Source: BMC Health Serv Res. 2017 Jun 19;17:410. doi: 10.1186/s12913-017-2359-0 (PMC5477151; doi:10.1186/s12913-017-2359-0)
Supplement: Supplementary file 6 — Italian Cancer Patients Education Group(ICPEG)_investigators research. (DOCX 10 kb) [file 12913_2017_2359_MOESM6_ESM.docx]

ICPEG, Italian Cancer Patient Education Group, include the following members participating in this study are: Marilena Bongiovanni, President of the ANGOLO-National Association of Oncology Survivors Patients Onlus; Marta De Conti, ANGOLO Onlus; Francesca Agostina Masutti, ANGOLO Onlus; Silvia Flora, Radiotherapy, CRO Aviano National Cancer Institute - IRCCS, Aviano; Giulia Fornasier; Pharmacy Clinical Desk , CRO Aviano National Cancer Institute, Aviano; Sara Francescon, Pharmacy Clinical Desk, CRO Aviano National Cancer Institute, Aviano; Valentina Tomasi, Press Office, Azienda Ospedaliero-Universitaria-Ferrara; Carla Bagnacani**,** Medical Library, Arcispedale Santa Maria Nuova - IRCCS, Reggio Emilia; Angela Nardi,Istituto Scientifico Romagnolo per lo Studio e la Cura dei Tumori (IRST) IRCCS, Meldola(FC); Cristina Nanni, Istituto Scientifico Romagnolo per lo Studio e la Cura dei Tumori (IRST) IRCCS, Meldola(FC); Petrina Mariana Braghesiu, Istituto Scientifico Romagnolo per lo Studio e la Cura dei Tumori (IRST) IRCCS, Meldola(FC), Italy
